# The Role of Sense of Voice Presence and Anxiety Reduction in AVATAR Therapy

**DOI:** 10.3390/jcm9092748

**Published:** 2020-08-25

**Authors:** Mar Rus-Calafell, Thomas Ward, Xiao Chi Zhang, Clementine J. Edwards, Philippa Garety, Tom Craig

**Affiliations:** 1 Mental Health Research and Treatment Center, Faculty of Psychology, Ruhr-Universität Bochum, 44787 Bochum, Germany; Xiaochi.Zhang@rub.de; 2 Department of Health Service and Population Research, Institute of Psychiatry, Psychology & Neuroscience, King’s College London, London SE5 8AB, UK; thomas.craig@kcl.ac.uk; 3 Department of Psychology, Institute of Psychiatry, Psychology & Neuroscience, King’s College London, London SE5 8AB, UK; thomas.ward@kcl.ac.uk (T.W.); clementine.edwards@kcl.ac.uk (C.J.E.); philippa.garety@kcl.ac.uk (P.G.); 4 South London & Maudsley NHS Foundation Trust, London SE5 8AZ, UK

**Keywords:** auditory hallucinations, voices, AVATAR therapy, anxiety, presence, exposure therapy

## Abstract

AVATAR therapy offers a unique therapeutic context that uses virtual reality technology to create a virtual embodiment of the voice-hearing experience, enabling the person to visualize their persecutory voice and engage in real-time “face-to-face” dialogue. The present study explores, for the first time, the contribution of sense of voice presence, together with session-by-session reduction of anxiety and paranoid attributions about the avatar, to changes in primary outcomes following AVATAR therapy. Data from 39 participants, who completed AVATAR therapy and attended a 12-week follow-up assessment, were analysed. Mid- to high-levels of sense of voice presence were reported across the therapy sessions, along with significant reductions of anxiety levels and paranoid attributions about the avatar. The interaction of sense of voice presence and reduction of anxiety was associated with two of the significant therapy outcomes: PSYRATS total and frequency of voices. The findings suggest that improvements in voice severity and frequency at post AVATAR therapy may be influenced by the combination of feeling less anxious in the context of a realistic simulation of the voice, while voice-related distress may involve additional cognitive and relational processes.

## 1. Introduction

Auditory verbal hallucinations (AVH), or the experience of hearing a voice without the corresponding external stimuli, can be understood as a communicative experience between the voice-hearer and their voice(s) [1]. A focus of recent research has been the individual’s perception of the voice, as coming from a characterised agent [2,3], with whom a personally meaningful relationship develops [4,5,6].

Recent developments in psychological therapies for distressing voices, especially relational approaches, have adopted the interpersonal relationship between the voice-hearer as their main intervention target [7,8,9]. In AVATAR therapy [10,11], a novel therapeutic context allows ‘face-to-face’ dialogue between the person and a computerised representation of their voice. Bespoke software transforms the therapists’ voice to match the pitch and tone of the chosen voice and the person creates a visual representation of their voice. The voice and image are combined to produce the ‘avatar’ (i.e., an animated talking virtual agent with enhanced lip-sync speech and eye blinking), through which the therapist interacts with the voice-hearer, in a three-way conversation (‘trialogue’). Where there are multiple voices, the participant selects one to work with (usually the most dominant, frequent and distressing). The aim is for the voice-hearer to develop an increased sense of power and control within the dialogue, consistent with broader cognitive approaches [12,13,14,15]. AVATAR therapy involves direct exposure to the anxiety-provoking fear stimuli (i.e., the representation of the voice and specific distressing content), providing an opportunity for the voice-hearer to face their voice within a controlled and safe space, and take back control over feared and disempowering experiences. The efficacy of the therapy has now been demonstrated in two independent pilot studies [9,16] and in a large fully-powered randomised controlled trial, comparing AVATAR therapy and Supportive Counselling: AVATAR therapy was found to be more effective post-therapy in terms of reductions in the frequency, distress and omnipotence of voices after an average of six therapy sessions [10].

Virtual reality has long been used to facilitate exposure to feared stimuli mostly for anxiety disorders (frequently referred as virtual reality exposure therapy or VRET) [17], with encouraging results for the treatment of psychosis [18]. Virtual environments can evoke responses in a participant that match those occurring in the natural environment. This offers a window into the user’s real-time behaviour, including interaction with virtual agents, and allows the person to test out new responses, for example, targeting the safety behaviours and paranoid attributions that can maintain voice-related distress [19,20,21]. Although AVATAR therapy, as originally developed by Leff and colleagues [9], is not delivered using a complex immersive environment, the platform uses virtual reality technology to create a virtual embodiment of the experience of hearing voices and to re-enact the relationship with the voice within real-time dialogue (see also du Sert et al. [16] for an independent pilot using a head mounted display to deliver the therapy). The embodiment of the voice is enhanced by the use of direct verbatim speech, and enactment of the ascribed character and background of the voice. Within the framework of embodied cognition, which holds that cognitive processes are rooted in the body’s interactions with the world [22], sense of presence refers to the individual’s psychological sensation of “*being there*” in the environment with the ability “*to do”* there [23]. Previous studies have shown people report feeling some level of presence in almost all computerised mediated environments [24]. This psychological phenomenon has been linked to knowledge transfer (i.e., skills or knowledge gained in virtual environment can be successfully transferred to the real world) [25], as well as possible enhancement of learning and performance [26]. This means that the more the virtual experience matches people’s real-world experiences, the more likely they will be able to generalize the learned responses to their daily life. Sense of presence has also been linked to clinical improvement when using mediated environments for treatment of mental health disorders. In particular, some studies have shown the reciprocal bi-directional relationship between presence and emotions: on one hand, the higher the presence, the higher the intensity of emotions experienced during the therapy and, on the other hand, the higher the intensity of emotions, the higher the sense of presence and reality judgment [27,28]. Relevant environments, providing emotionally significant content targeting specific emotions and related cognition for a specific population, and optimal induced levels of presence are required when delivering virtual reality based therapy. This can potentially result in greater clinical change [29].

In the context of working with voices, the AVATAR platform [9,10] creates a tailored mediated environment and delivers a realistic simulation of the experience of hearing and relating to the agent behind the voice. The traditional conceptualisation of sense of presence requires the introduction of two additional aspects: real-time communication and the enactment of the relationship between the person and their persecutory voice. This sense of voice presence supports in vivo cognitive and emotional work, focusing on associated meanings with the voice experience (e.g., paranoid beliefs). The AVATAR therapy structure involves a gradual change of the avatar’s character from hostile to conciliatory, targeting distressing beliefs and paranoid attributions associated with the voice, with a view to real-world generalisation.

Although the effectiveness of AVATAR therapy is now supported by several studies, there has not yet been any study exploring the impact of virtual embodiment of the hearing voice experiences and associated emotional and cognitive processes on therapy outcomes. The present study is the first exploring the sense of voice presence in a psychological therapy for distressing auditory hallucinations. More specifically, it analyses the contribution of sense of voice presence, together with a reduction in anxiety and paranoid attributions about the avatar, to significant primary therapy outcomes following AVATAR therapy. In line with the therapeutic targets of AVATAR therapy and main findings of the RCT [10,30], we hypothesised that; (1) the level of sense of voice presence would remain consistently high across the sessions; (2) there would be a significant reduction of anxiety and paranoid attributions across therapy sessions; and (3) better therapy outcomes at 12-week follow-up (as reported in Craig et al., 2018: PSYRATS-total, frequency, distress and BAVQ-R omnipotence) would be associated with; (i) sense of voice presence, (ii) anxiety reduction, (iii) decreased paranoid attributions to the avatar, as well as (iv) the interaction between anxiety reduction and sense of voice presence.

## 2. Methods

This study formed part of the AVATAR trial (ISRCTN65314790), a randomised clinical trial based in London (UK) which commenced in November 2013. The additional research ethics approval for the study reported here was granted in May 2014 (London-Hampstead Research Ethics Committee, reference 13/Lo/0482).

### 2.1. Participants:

The sample consisted of all participants from May 2014 allocated to AVATAR therapy who completed therapy and 12-week follow-up assessments. The inclusion criteria were as follows: (1) Aged over 18 years; (2) have experienced troubling auditory hallucinations for at least 12 months and (3) primary diagnosis of non-organic psychosis (including ICD-10 categories F20-29 and F30-39, subcategories with psychotic symptoms). Criteria for exclusion were as follows: (1) CBT for psychosis or attending a group specific to hearing voices; (2) unable to identify a single dominant voice to work on; (3) refusing all medication; (4) a diagnosis of organic brain disease; (5) a primary substance dependency; (6) auditory hallucinations not in English; (7) not having sufficient English language abilities to engage in therapy and assessments, and; (8) inability to tolerate the assessment process.

After providing informed consent, participants completed assessment of auditory hallucinations and other psychosocial processes with blinded and trained assessors at baseline, 12- and 24-week follow-up. Outcomes at 12-week follow up were used for analysis in the present study. The in-session measures for the present study formed part of the therapy booklet in the AVATAR therapy arm and were completed with participants following the active avatar dialogue each session (from session 1 until final attended session). AVATAR therapy consisted of one introductory session, which included creation of the avatar and comprehensive assessment of the voice(s), followed by six weekly 50-min sessions.

### 2.2. Measures

#### 2.2.1. Taken from Baseline and Follow-Up Assessments (Blind Assessors)

Psychotic Symptom Rating Scales—Auditory Hallucinations, (PSYRATS-AH [31]): The PSYRATS-AH is a semi-structured interview that consists of 11 items which measure different dimensions of auditory hallucination (frequency, duration, location, loudness, disruption, amount and intensity of distress, beliefs about origins, amount and degree of negative content, controllability). The PSYRATS has good inter-rater reliability (coefficients ranged from 0.88 to 1.00) and high internal consistency [31]. Following the dimensions identified by Woodward and colleagues [32] of the PSYRATS-AH and presentation of results of the AVATAR RCT, the total score and two dimensions of the PSYRATS-AH were used in the present study: Distress (grouping items of negative content, distress and control) and frequency (grouping items of frequency, duration, and disruption).

The revised version of the Beliefs about Voices Questionnaire (BAVQ-R, [33]) is a self-reported measure, which focuses on the person’s beliefs about voices as well as emotional and behavioural responses. In addition to the total score, there are three subscales relating to beliefs: malevolence, benevolence and omnipotence (six items per each subscale); and two further subscales that measure emotional and behavioural responses to the voices: resistance (9 items) and engagement (8 items). As per hypothesis three, only the subscale omnipotence was included in the analysis of the present study. All responses are rated on a four-point scale (0-disagree 1-unsure, 2-agree slightly, 3-agree strongly). The BAVQ-R is a valid scale to measure beliefs and emotions related to voices and has good internal consistency (α = 0.86, range from 0.74 to 0.88 for the 5 subscales) [33].

#### 2.2.2. In-Session Measures (Self-Reported by Participants)

State Social Paranoia Scale (SSPS adapted from [34]): This self-reported questionnaire was constructed originally to assess thoughts in relation to the virtual agents during the experience of a virtual environment. It consists of 20 items rated on a 5-point-scale, with 10 items measuring persecutory thoughts and 10 items assessing neutral and positive thoughts. For the purpose of this study, the statements/ thoughts were adapted to be answered in relation to the avatar (this adapted version was reviewed by main author of the scale Professor Daniel Freeman). Only the subscale on persecutory attributions was used for analyses. Examples of the items were as follows: *the avatar had bad intentions towards me, the avatar wanted me to feel threatened, the avatar was trying to intimidate me.*

Sense of Presence Scale (SUS questionnaire, adapted from [25]). This questionnaire consists of 6 items each rated on a scale of 1 to 7, with higher numbers indicating greater reported presence. The SUS questionnaire has shown good internal consistency (α = 0.75) [35]. For the present study, three of these six items were selected, and the Likert scale was adjusted to 1–5 to be consistent with the other in-session measures (i.e., SSPS and VAS). All items were summed in order to categorise the sense of voice presence with 1–5 = Low level of presence; 6–10 = Mid level of presence; 11–15 = High level of presence): *(1) How like your normal experience of hearing your troubling voice was your experience with the AVATAR, on a scale of 1 to 5, where 5 represents your normal experience of hearing this voice? (2) When you think back to the experience with the avatar, do you think of it more as an image that you saw or more as your voice talking to you? (3) During the time of your experience, how often did you think to yourself that you were actually hearing your troubling voice?*

Anxiety visual analogical scale (VAS): Following the dialogue participants were asked to rate the level of anxiety they experienced in dialogue with the avatar from 1 (not at all) to 5 (very anxious).

### 2.3. AVATAR Therapy

AVATAR therapy involves two phases: Phase 1 (sessions 1 to 3) focus on exposure and assertive responding; Phase 2 (sessions 4 to last) focus on relational, emotional and developmental processes [21,30]. It offers a unique way for clinicians to understand how voice hearers interact with a realistic representation of their voice. By switching between voicing the avatar and speaking as themselves, the therapist facilitates an interaction in which the participant gains a sense of power and control as the avatar changes from domineering and persecutory towards more supportive and conciliatory. Early sessions involve the voice-hearer standing up to the avatar who delivers distressing verbatim voice-content (“*I’m taking you to a dark place*” “*You are a worthless*”) in a characterised manner voiced to match the person’s description of the voice.

### 2.4. Statistical Analyses

Data analysis was carried out using SPSS for Windows (version 26.0, 2019). Descriptive analyses were used to describe reduction of anxiety and paranoid thoughts over the six active therapy sessions, and repeated measures t-test differences were performed to compare self-reported anxiety, sense of presence and paranoid attributions between Session 1 and the final attended session (typically Session 6). Multiple linear regressions were used to analyse the association of better therapy outcomes (T_12-weekFU_—T_Baseline_) with sense of presence (average score over 6 sessions), reduction of anxiety (change between session 1 to last session), reduction of paranoid attributions (change between session 1 to last session) and the interaction between Sense of voice presence*Anxiety reduction. Results were considered significant at *p* ≤ 0.05 and effect sizes (Cohen’s d) were reported.

## 3. Results

### 3.1. Demographics

Forty-four participants were eligible for the study (having completed therapy), 2 people did not complete the in-session questionnaires (incomplete data for 3 or more sessions) and 3 people completed the therapy but did not attend the 12-weeks follow-up assessment (please see Craig et al. [10] for total number of randomised participants and drop-outs in AVATAR therapy in the main RCT). Demographic and clinical data for the final sample (*n* = 39) are summarised in Table 1.

Thirty-eight participant attended the standard 7 sessions of AVATAR therapy (including the initial session of assessment and avatar creation), and one participant was considered a completer after 3 sessions of AVATAR therapy after reporting complete absence of voices for more than two weeks.

### 3.2. Hypothesis 1 Sustained Levels of Sense of Voice Presence Across Therapy Sessions

Mid to high levels of sense of presence (> 10) were reported consistently across the therapy sessions (no significant difference between last session cf. session 1, *p* = 0.10, Cohen’s d = 0.2), indicating that the avatar dialogue was effective in its aim of delivering a valid simulation of the voice heard by the person during everyday life.

### 3.3. Hypotheses 2 Reduction of Anxiety and Paranoid Attributions Across Therapy Sessions

**Figure 1:**
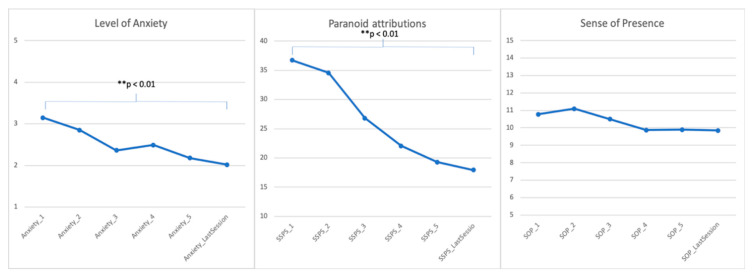
Levels of anxiety, paranoid attributions to the avatar and sense of voice presence (SOP) across therapy sessions.

Statistically significant reductions in self-reported levels of anxiety and paranoid attributions were observed between session 1 and the last session of therapy (see Figure 1). An exploratory data analysis was also carried out to look at early anxiety reduction (during phase 1: sessions 1 to 3) and late anxiety reduction (during phase 2: sessions 4 to last) (see Table 2). Results indicate a significant reduction during Phase 1 but no change in Phase 2; this pattern is consistent with the planned delivery of therapy across these two phases.

### 3.4. Hypothesis 3 Associations with Better Therapy Outcomes

Table 3 shows mean and standard deviations of the significant primary therapy outcomes for the current sample, as well as the average improvement (difference scores between baseline and those at 12-weeks follow-up).

The proposed model was significant for two of the therapy outcomes: PSYRATS total and frequency of voices, explaining 24% and 28% of the total variance, respectively.

Improvement in total PSYRATS and frequency of voices was related to only one predictor: the interaction between anxiety and sense of presence of both PSYRATS total (t_reduction_anxiety * sense_presence_ = 2.39, = 0.47, *p* = 0.02) and PSYRATS frequency subscale (t_reduction_anxiety * sense_presence_ = 2.94, = 0.47, *p* < 0.001).

None of the hypothesised predictors had an effect on improvement of voice-related distress (PSYRATS-AH distress) or omnipotence beliefs about the voices (BAVQ-R Omnipotence) (Table 4).

## 4. Discussion

The present study is the first to explore the sense of voice presence in a psychological therapy for voices and to investigate the impact of voice presence, reduction of anxiety and paranoid attributions on AVATAR therapy outcomes. Our results show, firstly, that sense of voice presence remained high over the course of the therapy, indicating that the avatar dialogue was effective in its aim in providing a valid simulation of the voice heard by the person during everyday life. Consistent with the explicit AVATAR therapy treatment aims, levels of self-reported anxiety and paranoid attributions about the avatar reduced significantly over the course of therapy. However, a sense of voice presence, reduction in anxiety and paranoid attributions did not individually predict any treatment outcomes. Rather, only the interaction effect between sense of presence and anxiety reduction was associated with better outcomes at post-therapy, specifically the PSYRATS total and frequency of voices.

The study provides evidence for the delivery of a consistent sense of voice presence, which is viewed as important to the successful AVATAR therapy delivery. This allows the creation of a unique and flexible mediated environment, maintained by the evolving dialogue with an avatar voiced by the therapist to mirror the characterisation and relational style of the everyday voice [21]. The use of technology facilitates three important facets of AVATAR therapy: (1) The incorporation of a tangible “self” or “other” representation, helping to differentiate between the two agents of the relationship; (2) improvement of the interactivity, realism and impact of the dialogue between the person and the agent behind the voice, and (3) reducing cognitive and behavioural avoidance, during exposure to the voice as fear stimuli. Establishing the sense of voice presence starts prior to the first dialogue, with the process of creating the avatar, and matching the auditory characteristics and associated image to the person’s main voice. Each avatar was unique to the individual, with the bespoke AVATAR software allowing the therapy to reflect the diversity of the voice-hearing experience [3,5]. Creating and interacting with the avatar acts as a powerful validation of the voice-hearer’s experience, indeed many participants commented that the therapists were the first to hear the voice as they experienced it. By seeing and hearing the voice in real time, the AVATAR approach facilitates the activation of fear and offers access to emotion and emotional meaning-making (‘hot’ cognition), and the potential for meaningful change during what is a comparatively brief therapy [30].

The key finding of the effect of the interaction between sense of voice presence and anxiety reduction on therapy outcomes, specifically PSYRATS-AH total and frequency, is important to understanding the processes occurring within the AVATAR therapy context. This result is in line with the results reported mainly in the field of anxiety disorders [28] and suggests that meaningful clinical changes after AVATAR therapy rely on providing a realistic simulation of the voice experience capable of triggering and reducing the targeted emotions, in particular fear and anxiety. Effective exposure in a “safe space” allows the person to drop safety behaviours (e.g., avoidance to talking back or looking at the avatar), which may be crucial for attenuating the conditioned feared response. By replying assertively to the avatar, the person experiences the non-occurrence of the expected negative outcome (expectancy violation, ‘nothing bad happens’). In traditional exposure therapy, emotional processing is proposed as a mechanism of change, with the necessity of a fear structure to be activated [36]. The activation of fear, in this case occurring under optimal therapist controlled conditions of a sensed voice presence, may facilitate new and corrective information to be incorporated in the cognitive structures (including memory) and leads to a change in fear response [37]. Learning to face this potentially terrifying presence and overcoming the initial fear reaction in a safe and supported way, can be a crucial step towards changing the relation to a voice [30].

Typically, the results from controlled trials of psychological interventions for auditory hallucinations show significant effects on appraisals of voices and related distress [38]. AVATAR therapy is one of the few psychological therapies that, in addition to reduction of distress and impact on omnipotent voice appraisals, has shown an effect on reduction on frequency of voices. As discussed above, reduced voice frequency appears to be associated with the interaction between sense of presence and anxiety reduction during therapy sessions. Contrary to our expectations, we found no significant effects of voice presence, reduction of anxiety, their interaction and paranoid attributions on post-therapy reductions in voice-related distress or omnipotence, suggesting that these outcomes may relate more strongly to other processes targeted within AVATAR therapy. Consistent with relational and cognitive models [13,14,39,40], the strategies used in AVATAR therapy, which focus on relational, developmental and emotional processes may be more influential in observed changes in the distress and omnipotence beliefs. These therapeutic elements include improvement of self-esteem, working towards internal attributions, re-building self-identity and social inclusion, compassion to the voice, experiential disengagement or work on grief and trauma [21,30]. The absence of an association between reduction in paranoid attributions to the avatar on therapy outcomes suggest that this reduction may be primarily attributable to the avatar becoming more conciliatory as a planned part of the therapy process.

The exploratory analysis of anxiety reduction during the two therapy phases indicated a significant reduction during Phase 1 (as would be expected from the early focus on exposure and assertiveness), but no change in Phase 2, during which the avatar has transitioned to a more conciliatory position. One question emerging from these preliminary results is whether a stand-alone “6-sessions phase 1” could be sufficient in those cases where distress and interference of the voices on the person’s daily life are closely linked to anxiety and fear. A rapid reduction of anxiety could be consolidated with an increased focus on exposure to verbatim content, combined with essential components of assertiveness and self-esteem. This approach could be standardised in order to reduce the complexity of therapy delivery and reliance on highly experienced therapists, which would, in turn, facilitate wider dissemination. In contrast, an extended version of the therapy including an expanded phase 2 introducing further personalisation and optimisation of the therapy could be of particular benefit for those with more complex relationship/highly characterised or trauma-related voices [7,39]. We intend to explore this further in future work.

The findings of the present study need to be understood in the context of several limitations. Firstly, the results refer to only one therapy group (AVATAR therapy arm) with the implication that there is no possibility to perform a mediation or moderation analysis with a comparison group. Secondly, the in-sessions measures are self-reported and both the SSPS and SUS required adaptation to the current therapy approach (i.e., referring to the avatar). Future research should include objective measures of anxiety including physiological arousal (e.g., heart rate variability or skin conductance) and/or other methods of measuring anxiety involving changes in tone or prosody of the person’s voice. Thirdly, while it offers a significant novel contribution to our understanding of AVATAR therapy, the concept and measurement of sense of voice presence requires further development. Other important aspects of the phenomenology of voices may have impacted on the findings of the study: the level of characterisation of the voice could have contributed to a higher sense of voice presence, while content of the dialogue could have influenced distress when conversing with the avatar. Finally, the current results will be complemented by a qualitative investigation of people’s experience of taking part in AVATAR therapy and further exploration of their views on voice embodiment and emotional reactions (e.g., shame, anger or guilt) during therapy. Larger fully powered studies are required to replicate these findings, potentially including other relevant predictors of change in therapy.

## 5. Conclusions

In the context of AVATAR therapy, the exposure to a virtual audio-visual representation of the voice matching the chosen auditory hallucination establishes a sense of voice presence, which in combination with anxiety reduction, contributes significantly to the overall improvement and reduction of voice frequency. Hence, the success of certain therapy outcomes relies on bringing the voice “online” during therapy, with implicit activation of structures related to fear, and consequent desensitisation (i.e., reduction of anxiety). Other improvements in voice-related distress may be more associated with aspects of AVATAR therapy not assessed in current study, e.g., relational and developmental work on meaning-making and re-calibration of the interpersonal relationship with the voice. It remains an open empirical question whether these processes might also interact with sense of voice presence in order to influence outcome. AVATAR therapy is a unique intervention, delivering a personalised and tailored interactive experience to the voice-hearer. In the future, other forms of technology, such as immersive social environments or smartphone apps, used in combination with AVATAR therapy, could expand the therapeutic targets and promote the generalisation of therapy improvements to the voice-hearers daily life and social relationships.

## Figures and Tables

**Table 1:** Demographics and clinical data (N = 39).

	N (%)	M (SD)
**Age**		43.87 (9.33)
**Gender**		
Male	30 (77%)	
Female	9 (23%)	
**Ethnicity**		
White British	13 (33%)	
Black British	5 (13%)	
Black Caribbean	3 (8%)	
Black African	5 (13%)	
Asian Indian	3 (8%)	
Other	10 (25%)	
**Education**		
Primary	3 (8%)	
Secondary or equivalent	18 (46%)	
Vocational education	9 (23%)	
University degree	9 (23%)	
**Employment ^a^**		
Employed full-time	0 (0%)	
Employed part time	3 (8%)	
Unemployed	36 (92%)	
Student	2 (5%)	
Housewife/husband	1 (3%)	
**Marital Status**		
Married/cohabiting	1 (3%)	
Single	30 (77%)	
Single in relationship	3 (8%)	
Divorced	5 (12%)	
**Diagnosis**		
Paranoid Schizophrenia	29 (74%)	
Schizoaffective Disorder	6 (15%)	
Unspecific Psychosis	1 (3%)	
Dep w/ psychotic symptoms	3 (8%)	
	**N**	**M (SD)**
**Number of voices**		
1	10 (25%)	
2	7 (18%)	
3	7 (18%)	
4	1 (3%)	
5	9 (23%)	
Unsure/many	5 (13%)	
**Length_Illness (years)**		21.79 (11.04)

^a^ These categories are not mutually exclusive.

**Table 2:** Levels of anxiety, sense of presence and paranoid attributions by session and differences between session 1 and last.

	Session_1	Session_2	Session_3	Session_4	Session_5	Session_Last	*t*_S1-SLast_	*p*	Cohen’s d
	Mean (SD)								
Anxiety	3.15 (1.18)	2.85 (1.33)	2.36 (1.09)	2.49 (1.25)	2.18 (1.14)	2.03 (1.16)	4.29	*p* < 0.001	0.7
Anxiety S1–S3							3.33	*p* < 0.001	0.6
Anxiety S4–SLast							0.97	0.33	0.2
Sense of Presence	10.78 (2.61)	11.10 (2.48)	10.49 (2.76)	9.87 (3.05)	9.90 (3.28)	9.85 (3.21)	1.70	0.10	0.2
Paranoid Thoughts	36.72 (9.32)	34.54 (10.38)	26.82 (12.85)	22.06 (14.54)	19.26 (12.23)	17.95 (12.64)	9.15	*p* < 0.001	1.4

*Note*: S1: Session 1; S3: Session 3; SLast: Last session.

**Table 3:** Means and standard deviations at baseline, 12-weeks follow up and improvement for significant therapy outcomes (N = 39).

	Baseline M (SD)	12-Week FU M (SD)	Improvement M (SD)
PSYRATS-AH_TOTAL	27.87 (4.61)	21.41 (9.5)	6.46 (8.52)
PSYRATS-AH_Frequency	6.33 (2.08)	4.87 (2.55)	1.46 (2.01)
PSYRATS-AH_Distress	14.75 (2.97)	10.38 (5.52)	4.35 (5.32)
BAVQ-R_Omnipotence	9.87 (3.99)	6.79 (4.51)	2.05 (3.81)

*Note*: PSYRATS-AH: Psychotic Symptom Rating Scales-Auditory Hallucinations; BAVQ-R: Beliefs about Voices Questionnaire-Revised.

**Table 4:** Results of linear regression analysis by significant therapy outcome.

	*t*	Beta	*p*	R^2^	F	df	*p*
**Improvement in PSYRATS-AH (Total)**							
Overall model				0.24	2.62	4	0.04 *
Reduction of Anxiety	−52	−0.07	0.61			1	
Sense of Presence	0.93	0.14	0.35			1	
Reduction of Paranoid Attributions	0.25	0.03	0.80			1	
Reduction of Anxiety * Sense of Voice Presence	2.39	0.46	0.02 *			1	
**Improvement in PSYRATS-AH (Frequency)**							
Overall model				0.28	3.02	4	0.03 *
Reduction of Anxiety	−0.66	−0.08	0.51			1	
Sense of Presence	0.56	0.07	0.64			1	
Reduction of Paranoid Attributions	1.48	0.16	0.14			1	
Reduction of Anxiety * Sense of Voice Presence	2.94	0.47	0.00 **			1	
**Improvement in PSYRATS-AH (Distress)**							
Overall model				0.09	0.80	4	0.53
Reduction of Anxiety	−0.62	−0.41	0.53			1	
Sense of Presence	0.46	0.05	0.65			1	
Reduction of Paranoid Attributions	0.61	0.00	0.54			1	
Reduction of Anxiety * Sense of Voice Presence	1.67	0.37	0.10			1	
**Improvement in omnipotence BAVQ-R**							
Overall model				0.04	0.36	4	0.83
Reduction of Anxiety	0.26	0.05	0.26			1	
Sense of Presence	0.39	0.08	0.69			1	
Reduction of Paranoid Attributions	−0.87	0.15	0.39			1	
Reduction of Anxiety * Sense of Voice Presence	0.51	0.13	0.61			1	

*Note:* PSYRATS-AH: Psychotic Symptom Rating Scales-Auditory Hallucinations; BAVQ-R: Beliefs about Voices Questionnaire-Revised. * *p* < 0.05; ** *p* < 0.01.

## Data Sharing

According to Wellcome Trust’s Policy on data, software and materials management and sharing, all data supporting this study will be available in anonymised form from the corresponding author (MRC) on reasonable request, subject to review, following the publication of results.
